# Light blocking film in a glasshouse impacts *Capsicum annuum* L. yield differentially across planting season

**DOI:** 10.3389/fpls.2023.1277037

**Published:** 2023-12-19

**Authors:** Chelsea R. Maier, Sachin G. Chavan, Norbert Klause, Weiguang Liang, Christopher I. Cazzonelli, Oula Ghannoum, Zhong-Hua Chen, David T. Tissue

**Affiliations:** ^1^ National Vegetable Protected Cropping Centre, Western Sydney University, Penrith, NSW, Australia; ^2^ Hawkesbury Institute for the Environment, Western Sydney University, Penrith, NSW, Australia; ^3^ School of Science, Western Sydney University, Penrith, NSW, Australia; ^4^ Global Centre for Land-Based Innovation, Western Sydney University, Penrith, NSW, Australia

**Keywords:** light blocking film, protected cropping, energy use, light quality, light intensity, resource sustainability, *Capsicum annuum* L., agricultural technology

## Abstract

High energy costs are a barrier to producing high-quality produce at protected cropping facilities. A potential solution to mitigate high energy costs is film technology, which blocks heat-producing radiation; however, the alteration of the light environment by these films may impact crop yield and quality. Previous studies have assessed the impact of ULR 80 [i.e., light-blocking film (LBF)] on crop yield and photosynthetically active radiation (PAR); however, an assessment of the spectral environment over different seasons is important to understand potential crop impacts through different developmental phases. In this study, two varieties (red and orange) of *Capsicum annuum* were grown across two crop cycles: one cycle with primary crop growth in the autumn (i.e., autumn experiment [AE]) and the other with primary crop growth in the summer (i.e., summer experiment [SE]). LBF reduced PAR (roof level: 26%–30%, plant canopy level: 8%–25%) and net radiation (36%–66%). LBF also reduced total diffuse PAR (AE: 8%, SE: 15%), but the diffuse fraction of PAR increased by 7% and 9% for AE and SE, respectively, potentially resulting in differential light penetration throughout the canopy across treatments. LBF reduced near-infrared radiation (700 nm–2,500 nm), including far-red (700 nm–780 nm) at mid- and lower-canopy levels. LBF significantly altered light quantity and quality, which determined the amount of time that the crop grew under light-limited (<12 mol m^−2^ d^−1^) versus sufficient light conditions. In AE, crops were established and grown under light-limited conditions for 57% of the growing season, whereas in SE, crops were established and grown under sufficient light conditions for 66% of the growing season. Overall, LBF significantly reduced the yield in SE for both varieties (red: 29%; orange: 16%), but not in AE. The light changes in different seasons in response to LBF suggest that planting time is crucial for maximizing fruit yield when grown under a film that reduces light quantity. LBF may be unsuitable for year-round production of capsicum, and additional development of LBF is required for the film to be beneficial for saving energy during production and sustaining good crop yields in protected cropping.

## 1 Introduction

Researchers worldwide have attempted to reduce energy use in protected cropping (PC) food production (Ali & Albayati, 2017). In some PC facilities, such as high-tech greenhouses, wavelength-selective photovoltaics have replaced glass paneling in glasshouses to capture energy from less biologically relevant wavelengths of the light spectrum to offset energy consumption, while allowing the most important wavelengths to be utilized by the crop (Loik et al., 2017; He et al., 2021). Dye-sensitized and opaque photovoltaics have been mounted on greenhouses, which shade the crop but also produce electricity (Ntinas et al., 2019; Yano and Cossu, 2019). Recently, a radiation-reducing film (light-blocking film; LBF) developed for residential buildings to reduce the transmittance of heat-producing light has been used; hence, it might be useful for reducing the energy costs associated with crop production (Chaiyapinunt et al., 2005; Chavan et al., 2020). Although wavelength-selective photovoltaics, opaque photovoltaics, and LBF can offset or reduce energy expenditure and water and fertilizer use, they may also impact the quantity and quality of the light for crop production Chavan et al., 2020; Loik et al., 2017; Yano and Cossu, 2019; Zhao et al., 2021; Lin et al., 2022). Hence, it is necessary to understand how crops respond to these changes in light quality and quantity and how energy-saving LBF can be optimized to achieve more sustainable food production in greenhouses in the future.

Light quality and quantity affect plant development, physiology, and yield (Trouwborst et al., 2010; Bugbee, 2016; Poorter et al., 2019; Zhen and Bugbee, 2020). The spectral distribution of solar radiation can be described as a continuous range of wavelengths: ultraviolet radiation (UV: 200 nm–400 nm; about 5% of global solar radiation), photosynthetically active radiation (PAR: 400 nm–700 nm; about 45%), and near infrared radiation (NIR: 700 nm–2,500 nm; about 50%) (Abdel-Ghany et al., 2012). Each of these regions has been associated with varied effects on plant development (Kami et al., 2010; Kendrick and Kronenberg, 2012).

Many studies have investigated the light spectral impacts on crop performance and growth using monochromatic light (Azad et al., 2011; Lin et al., 2013; O’Carrigan et al., 2014), which are narrow-band spectral regions of light used to measure plant photosynthetic responses. Previous studies have considered photosynthetic activity using monochromatic LED lights that would produce specific bandwidths of light and found that photosynthetic activity drops at wavelengths >700 nm (Zhen and Bugbee, 2020). However, more recent studies have found that measurements conducted under a broader spectrum of light (400 nm–725 nm) increased CO_2_ assimilation (photosynthesis) by 10%–21%, suggesting that the impact of photon flux density was underestimated in the past (Zhen and Bugbee, 2020). These broader spectra are important, but these studies do not provide information on the quantity of light at each wavelength, are not easily quantifiable in greenhouses and are relatable to plant developmental responses. Scientists and researchers have not yet agreed on the numerical parameters for light quality, and more research is needed, with particular attention paid to the continuous measurement of light quality throughout the plant life cycle (Azad et al., 2011; Casierra-Posada et al., 2014).

Variations in natural light also affect plant development (Dokoozlian and Kliewer, 1996). Daily total natural light variation as measured by the daily light integral (DLI; mol photons m^−2^ d^−1^) from 400 nm to 700 nm is correlated with changes in plant physiology, development, and nutrient composition. For instance, a meta-analysis found that DLI was positively correlated with leaf mass per area, leaf thickness, and stomatal conductance and negatively correlated with specific stem length, total leaf nitrogen, and leaf area ratio (Poorter et al., 2019); however, light quality measurements were not considered in this meta-analysis. At similar DLI levels, the spectral quality profiles may be different in different scenarios. While spectral quality varies throughout the year with changes in the solar azimuth across solar transition periods (equinoxes and solstices) (Lean and Deland, 2012), spectral quality can also shift on consecutive days at the same time of the day if clouds are present. Cloud-immersed days could reduce the total solar radiation by up to 85%. In a study investigating light quality in the forests of the Appalachian Mountains, it was found that during cloudy days, blue light was enhanced by 5%–15% at the top of the forest canopy, while transmittance to the understory was reduced by 25%–60%. On cloud-immersed days, red light decreased by 6%–11%; however, transmission of red light to the understory increased by 25%–30%. These results, while in a forest setting, indicate that clouds impact spectral quality and quantity, as well as through-canopy transmission of specific wavebands (Reinhardt et al., 2010).

Crops behave differently, depending on the relative proportion of light reduction. Overall, a reduction in cumulative light resulted in a reduction in both fresh and dry weights. Herbs grown under colored film reduced total PAR by 34% and decreased herb dry weights by 29%–40% (Hückstädt et al., 2013). In the production of cut flowers, decreased radiation reduced the number of shoots, shoot weight, and quality of roses. It has been estimated that a 1% reduction in radiation will result in a 0.8%–1% reduction in yield, with lower radiation conditions having a relatively stronger impact during the low radiation months of winter compared to summer (Marcelis et al., 2006). For lettuce under step decreases in light intensity, Kosma et al. (2013) found that fresh weight was reduced significantly at each light intensity reduction for both the winter and spring seasons. Although there are seasonal light differences in DLI maxima, crops also behave differently depending on the photoperiod ascension or descension (Heuvelink, 1995).

The proportion of diffuse light also varies with time of day and external conditions, and impacts crop plant development and photosynthesis. Photosynthesis depends on both incident light and light penetration into the canopy mass, such that photosynthesis can be higher in lower PPFD under diffuse light conditions than under direct light conditions (Hemming et al., 2006; Markvart et al., 2010). Depending on the light conditions, hazed glass, especially with the addition of a topical film, may increase the diffuse light fraction received by the crop. On high-light days, 90% of light is intercepted within the upper 50%–60% portion of the crop canopy reducing the ability of lower leaves to contribute to photosynthesis, ultimately reducing assimilate supply and thus reducing fruit yield capacity. Interlighting with LEDs has been used to overcome the impact of shaded lower canopy regions and has been shown to increase fruit yield in capsicum (Jokinen et al., 2012). Within a glasshouse, large areas of shading occur because of the structural components of the facility and light is not well-distributed to the growing plants underneath (Gruda, 2005). Most high-tech glasshouses use high-quality hazed glass to reduce these shaded areas because the hazed glass further diffuses light upon transmission. Diffuse light is incident on more surface angles that are present within the canopy than direct light due to these multiple angle points, thereby increasing total crop photosynthesis (Hemming et al., 2008). Days with a high proportion of diffuse light can also increase the total radiation due to reflectance off clouds, allowing for higher light intensity and further light penetration throughout the canopy (Priva Help Center, 2022). LBF is a film applied to hazed glass that diffuses light incident on a crop; therefore, it is important to understand how LBF impacts the diffuse light environment in a glasshouse.

Spectral quality and quantity vary significantly over short- (minutes) and long-term (months) intervals and are the key factors affecting crop production. While there are numerous reports of light impacts on PC horticultural crops, much of the research is based in low light environments in Europe where most of the design of glasshouses takes place (Montagu, K. 2018). As such, glasshouses may not be optimized for the high-light Australian horticultural industry which experiences significantly higher radiation loads (Montagu, K. 2018). In Australia, energy consumption is the second-largest cost of PC after labor costs. Hence, it is vitally important to measure the light quantity and quality regimes in PC under Australian solar and climatic conditions. LBF reduces the heat load when applied to glasshouse roofs and sidewalls, but it alters the spectral quantity and quality of light, and has been shown to affect crop development and yield of *S. melongena* and *C. annuum* (Chavan et al., 2022; T. Lin et al., 2022). However, the impact of LBF on spectral quality has not been fully assessed across a crop’s lifetime nor has it been compared across different planting seasons.

Although we did not conduct an economic analysis of LBF in this study, energy costs are the second highest associated with PC production, highlighting the need to implement energy-saving techniques, products, and infrastructure in existing PC facilities (Barbosa et al., 2015). The manufacturer of LBF, Saint-Gobain, reports that energy savings with the use of their film can be up to 30% in industrial or residential settings (Solar Gard Saint-Gobain, 2023). While these estimates do not encompass the use of LBF in PC facilities, the expected energy savings from LBF are theoretically proportional to the reduction in SW radiation; however, there may be inhibitory impacts from heat transfer through convection (Chaiyapinunt et al., 2005). Reducing the energy costs for the PC industry would reduce operational costs and greenhouse gas emissions, both benefiting communities at large (Maraveas et al., 2023). Government incentives exist in Australia for the implementation of energy-saving techniques in agriculture (Grants and funding, 2023). As agricultural film technology is still developing, these government agricultural incentives are not specifically aimed at LBF-type technologies; however, adoption of energy-saving films in PC agriculture is likely to be high, as it has short-term economic benefits (Piñeiro et al., 2020). The sustainability of these products, such as longevity and the ability to be recycled, is vital to their entry into and continued use in the PC industry, as environmental impact is a key consideration among producers when adopting new technologies (Piñeiro et al., 2020).

In our study, we investigated the impact of LBF on light spectral quality and quantity, and whether this impacts the plant growth and fruit yield of *C. annuum*? We investigated the impact of LBF on light environments, including PAR, shortwave (SW) and longwave (LW) radiation, and diffuse light during two *C. annuum* crop cycles, and the impact of altered light under LBF on crop growth and yield.

## 2 Materials and methods

### 2.1 Plant materials

Capsicum is one of the top 10 vegetables by volume and the top 15 by value within Australia. While most capsicum are grown outdoors in Queensland, capsicum is increasingly grown in high-tech greenhouses year-round in Australia’s cooler southern states (Horticulture Innovation Australia, 2019). The LBF project targeted two varieties of *C. annuum* that were grown at the National Vegetable Protected Cropping Centre (NVPCC). The project consisted of two crop cycles: (1) crop grown starting in a low light (ascending photoperiod) environment (transplant date: 5 April 2019, removal date: 5 December 2019) using varieties Gina (red) and O06614 (orange); and (2) crop grown starting in a high light (descending photoperiod) environment (transplanting date:17 January 2020, removal date: 23 September 2020) using varieties Gina (red) and Kathia (orange). All the varieties were sourced from Syngenta Australia (Macquarie Park, NSW, Australia).

### 2.2 Description of the glasshouse facility, LBF film characteristics, and experimental design

The NVPCC was established jointly by Western Sydney University and Horticulture Innovation Australia in 2017 at the Western Sydney University Hawkesbury Campus, Richmond, NSW, Australia (latitude: −33.611692° S, longitude: 150.745281° E). The NVPCC utilizes an 1,800 m^2^ high-tech autonomous hydroponic glasshouse, based on facilities designed in the Netherlands, that is environmentally controlled by Priva software and hardware (Priva, De Leir, The Netherlands). It was established as a research, education, and training facility to address the most pressing horticultural research questions and train emerging leaders in the Australian PC industry.

The LBF experiment used four 105 m^2^ glasshouse research compartments. All the research compartment roofs were fitted with HD1AR 70% hazed glass, and the walls were fitted with tempered clear glass. Two of these compartments were used as the controls. The treatment (LBF) compartments had an LBF film, which is a ULR-80 window film (Solar Gard, Saint-Gobain Performance Plastics, Sydney, NSW, Australia) designed for office buildings to reduce incoming sunlight and energy used to cool the building. The manufacturer states that the film blocks spectral light in varying amounts as follows: ~88% infrared and far-infrared light from 780 nm–2,500 nm and >99% of ultraviolet (UV) light from 300 nm–400 nm. Overall, LBF blocks 43% of the total solar energy while allowing 40% transmission, 54% absorption, and 6% reflection. The film was applied to the ceiling, side walls, entry walls, and shared interior walls of the treatment compartments. Because of the infrastructure of the mechanical coolers set at the entry of each treatment compartment, LBF was not applied to the three ceiling panels per compartment and the top eave panels of each entry wall.

Two trials of two *C. annuum* varieties were grown under the LBF treatment and control. The first trial began in autumn and is denoted as AE (Autumn Experiment) herein. The second trial began in the summer and was denoted as SE (Summer Experiment). Capsicum seedlings were transplanted into 1 m-long Grodan Grotop Expert rockwool slabs (Roermond, Limburg, The Netherlands) with four plants per slab in the control and LBF compartments. Each gutter contained 10 slabs, for a total of 240 plants per room. Two weeks after transplantation, two stems from each plant were selected and trellised onto plastic strings supported by a high-wire system. Plants were grown according to commercial practices of hydroponic production of vegetables in greenhouses under non-limiting water and nutrient (EC: 2.5 dS m^−1^–3.0 dS m^−1^, pH: 5.0–5.5) conditions at [CO_2_] (489.6 μl l^−1^ and 476.6 μl l^−1^ daytime average), temperature (25.3/19.3 and 25.2/19.3°C day/night average), RH (74.2/72.9% and 74.2/77.5%, day/night average) and natural light for AE and SE, respectively (He et al., 2022). The environmental variables, including temperature, relative humidity, and CO_2_ concentration at canopy level, were monitored in all glasshouse compartments at 5-minute intervals. Data were stored using the Priva system.

### 2.3 Light quantity and quality measurements

A huge array of light sensors is available to characterise and quantify the spectrum of light received by plants. PAR sensors measure photon flux density in photons m^−2^ s^−1^ from 400 nm to 700 nm. Net radiometers measure incoming and outgoing LW and SW radiation in W m^−2^, and while SW and LW bandwidths differ slightly between instrument models, in general the bandwidth is 350 nm–2,500 nm for SW radiation and 2,500 nm–50,000 nm for LW radiation. Net radiometers were designed to measure the energy balance of a system and are thus important instruments for understanding energy fluxes (Mauder et al., 2020).

One major issue with most PAR sensors on the market is that they are usually calibrated for open-sky broad-spectrum solar radiation from 400 nm to 700 nm, and do not reflect PAR from monochromatic, supplemental, or spectrally altered light sources. Although PAR sensors measure total light from 400 nm to 700 nm they do not measure individual wavebands. Therefore, a spectroradiometer is necessary to understand the spectra available to the plant. Spectroradiometers measure instantaneous quantities of photons from higher resolution bandwidths (2 nm–15 nm resolution), usually within the 300 nm–1,200 nm range, and sometimes up to 2,500 nm, as is the case for the ASD FieldSpec (Malvern Panalytical, Malvern, Worcestershire, UK). The advantage of these sensors is that they measure the quantity of light of each wavelength incident on the crop, and these data can be transformed into PAR for comparability.

In August 2018, light sensor arrays were installed to characterize the light environment in both the control and LBF research rooms. These were connected to CR1000X data loggers (Campbell Scientific Inc., Logan, UT, USA) and programmed to measure continuously at 5-minute intervals. Each research room contained a PAR sensor (LI-190SZ Quantum Sensor, LI-COR, Lincoln, NE, USA), which measures photons from 400 nm to 700 nm, at the top of each bay. The incoming and outgoing SW radiation, and LW radiation were measured at the top of each glasshouse room using a net radiometer (SN-500, Apogee Instruments Inc., Logan, UT, USA). Using this net radiometer, the energy balance of each compartment was calculated, which is critical for understanding the impact of LBF on the light environment for biological responses as well as for cooling and heating energy use required to maintain optimal temperatures throughout the plant growth and production cycle. Diffuse PAR radiation was measured in one control room and one LBF treatment room using a diffuse light sensor (BF5 sunshine sensor; Delta T Devices, Burwell, Cambridge, UK). Variation in PAR incident on the crop canopy was measured by PAR sensors (LI-190R-SMV-50 Quantum Sensor, LI-COR, Lincoln, NE, USA) positioned at a maximum of 50 cm above the crop canopy and raised intermittently before being obscured by the growing plants. See **Table 1** for technical information and the position of the light sensor.

**Table 1:** Light sensor array description with associated technical data and position within the LBF and control compartments used across both AE and SE.

Sensor Type	Spectral Range	Units	Position in room	Trait
PAR	Incoming 400 nm–700 nm PAR/Visible	µmol m^−2^ s^−1^	1—Southwest 2—South mid 3—Southeast 4—Northwest 5—Northeast 6 – top of room	Photosynthesis
Diffuse Light	Incoming 400 nm–700 nm PAR/Visible + Diffuse Fraction	W m^−2^	1—top of room (1 LBF room and 1 C only)	Photosynthesis
Net Radiometer	Incoming + outgoing SW 295 nm–2685 nm UV, Visible, NIR LW 5,000 nm–30,000 nm Infrared and Far Infrared	W m^−2^	1—top of room	Energy balance
Spectroradiometer	Incoming 300 nm–1,100 nm UV, PAR, NIR	W m^−2^	1—handheld used to assess light penetration throughout canopy	Light quality

### 2.4 Light penetration measurements

**Figure 1:**
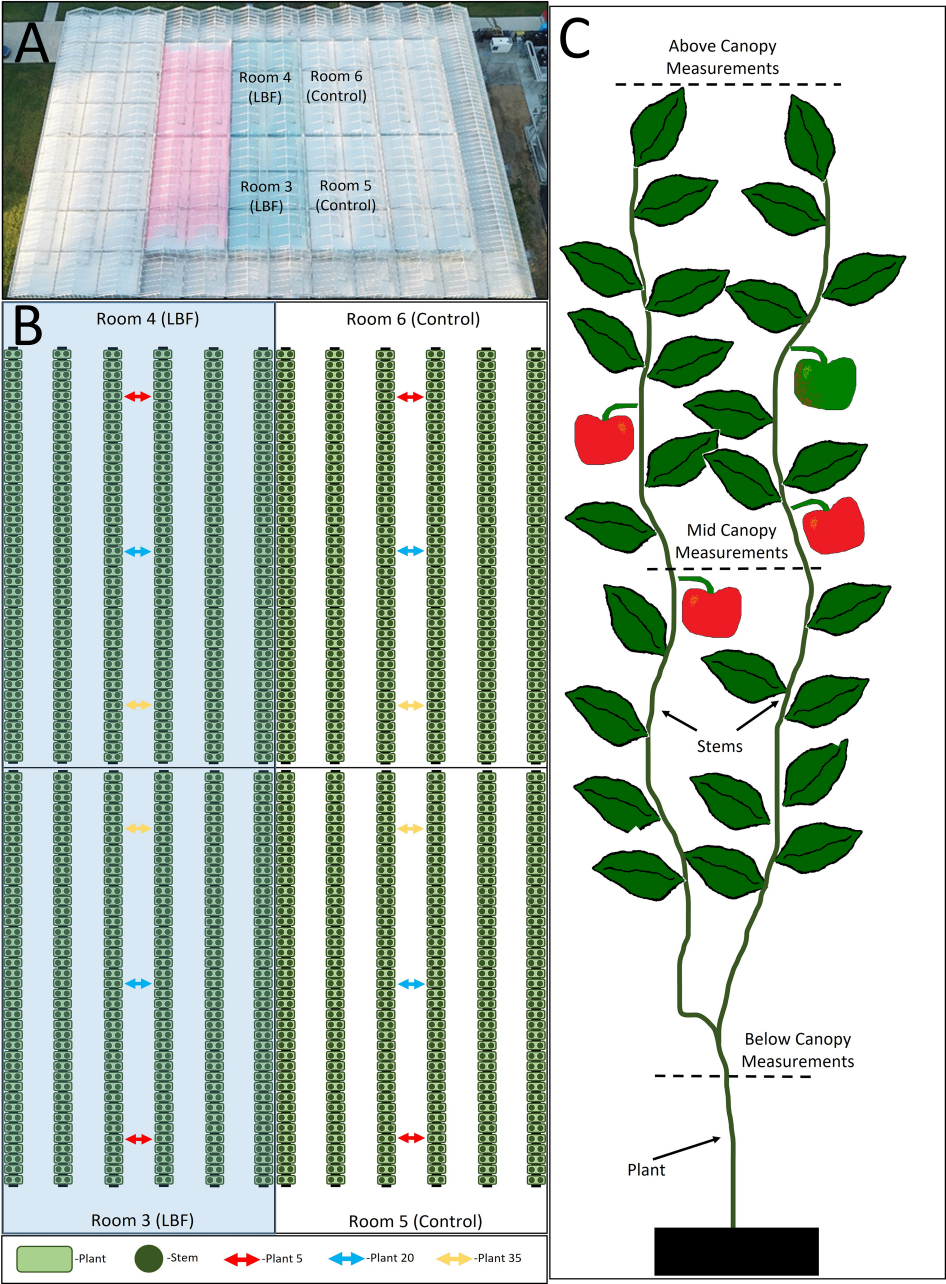
Diagram of glasshouse rooms and light penetration position measurements. Aerial view of the glasshouse with labeled research rooms **(A)**. Room diagram with indicated positions of plants 5, 20, and 35 where the light penetration measurements were performed **(B)**. Locations within the canopy light measurements were completed **(C)**.

To understand how light penetrated the crop canopy across the control and LBF, measurements were taken above the canopy, halfway down the canopy within the region of canopy growth (not the aisle), and at the base of the plant. Five measurements were taken at each canopy level at three positions along the gutter length of plants 5, 20, and 35 (**Figure 1**). These measurements were averaged per height for the LBF and control treatments.

### 2.5 Growth, yield, and biomass measurements

Growth was measured weekly, after two stems were selected from each plant. Plant growth rate was defined as the stem elongation rate in cm d^−1^. To measure this, the string supporting the stem of the plant was marked on the apical meristem of the stem. The length from the previous week’s mark to the current week’s mark was recorded. Pruning was conducted every two weeks. The pruned biomass was collected from 40 stems per variety. The material was dried at 70°C for a minimum of three days and weighed directly after cooling to room temperature. Counts of buds, flowers, and fruit (per 20 stems per variety per room) were conducted every two weeks before each scheduled pruning to assess differences in bud and flower presence and fruit carrying capacity across treatments.

Harvests of the capsicum crop were done weekly once fruits had ripened (90%–100% color change), and fruit number as well as individual weight were recorded for 40 stems per variety per room. The fruits were visually graded as follows: 1 if the fruit had a perfect shape, color, and shine; 2 if the fruit had a perfect color and shine but not a perfect shape; 3 if the fruit was misshapen with potentially some blemishes; and 4 if the fruit was tiny, diseased, and/or not edible. Grades 1 and 2 were selected to assess marketable yield and fruit number per plant.

Mature fruits, selected based on the color (red and orange) of individual fruits, were harvested, and the individual fruit weight and number of fruits per stem were recorded every week. The fruits were graded as marketable (≥100 g, including the extra-large fruit ≥250 g) and unmarketable, which included small (<100 g, edible) fruits and fruits with rotting, cracking, lobing, and other deformities.

### 2.6 Data analysis and statistics

All raw biological data were collated in Excel (version 2204, Microsoft, Redmond, WA, USA), and continuous environmental data were logged into CSV files and automatically saved. All data analyses were performed using the R software (R Core Team, 2021). PAR measurements were converted into daily light integrals (DLI; mol photons m^−2^ d^−1^) according to Poorter et al. (2019). Statistical analyses were performed following Chavan et al. (2020) because the crop measurement data and experimental design were similar. All statistical tests were performed using the R statistical package. The Shapiro–Wilks method was applied to verify whether data were normally distributed, and Bartlett’s test was used to verify the equality of variances. Once data were confirmed to be normally distributed with equal variance, one-way or two-way Analysis of Variance (ANOVA) was used. The Kruskal–Wallis Test was used when the data were not normally distributed but had equal variance. Welch’s ANOVA was used for normally distributed data with unequal variance. The p-values are either mentioned as values or as significance levels indicated as “*” (p-value <0.05), “**” (p-value <0.01) and “***” (p-value <0.001).

## 3 Results

### 3.1 LBF reduces PAR with a greater impact during high solar radiation conditions in both AE and SE

**Figure 2:**
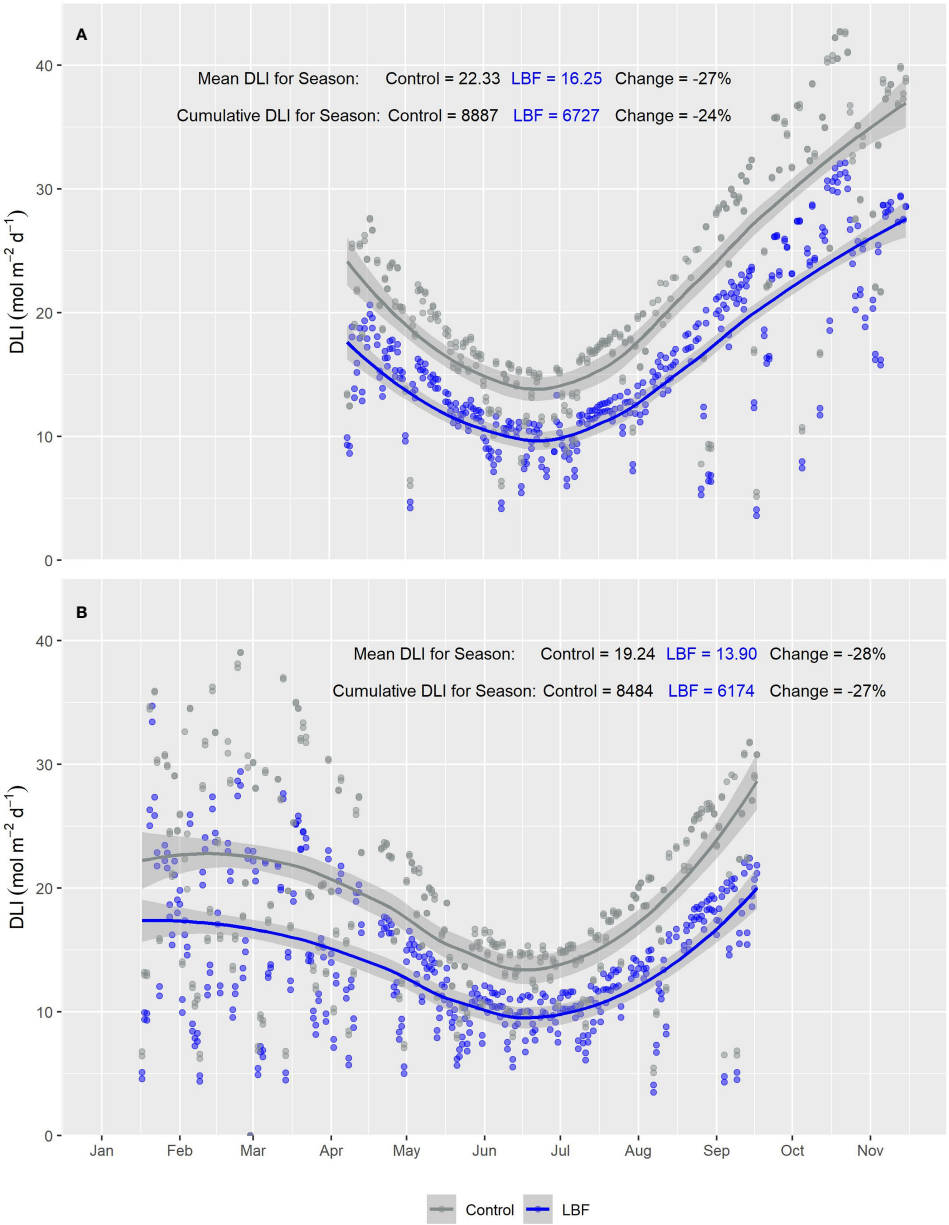
Smooth plot of the average cumulative daily light integral (DLI; mol m^−2^ d^−1^) over time for roof-level PAR sensors for both LBF and control for **(A)** Autumn Experiment (AE) and **(B)** Summer Experiment (SE). Each data point represents the average DLI for a single day. The blue (LBF) and gray (control) lines are fitted loess curves with formula y ~ x and shaded regions represent the 95% confidence interval.

**Figure 3:**
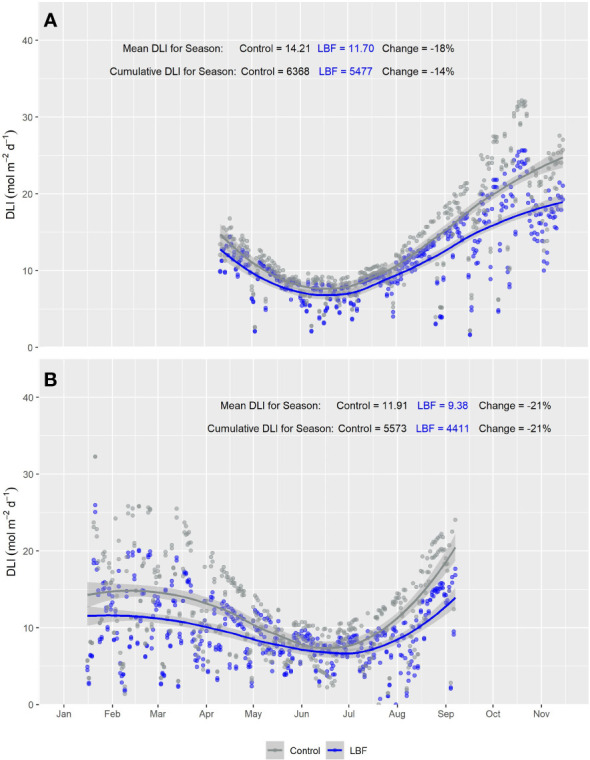
Smooth plot of cumulative daily light integral (DLI; mol m^−2^ d^−1^) over time across five canopy-level PAR sensors for both LBF and control treatments for **(A)** Autumn Experiment (AE) and **(B)** Summer Experiment (SE). Each data point represents the DLI for a single day across the five canopy-level PAR sensors. The curves are fitted with loess using the formula y ~ x. Blue (LBF) and gray (control) lines are fitted loess curves with formula y ~ x and shaded regions represent the 95% confidence interval.

The PAR sensors positioned at the top of each glasshouse room showed a consistent reduction in PAR across both AE and SE for LBF compared with the control. During AE, LBF reduced the mean DLI by 27% and cumulative DLI by 24% (**Figure 2A**), while during SE, LBF reduced the mean DLI by 28% and cumulative DLI by 27% (**Figure 2B**). However, for the canopy-level PAR sensors, the difference in PAR between the control and LBF was observed only in the high sun angle months of summer and when the canopy PAR sensors were at higher height positions throughout the seasons. Mean and cumulative reductions by LBF were observed for canopy level PAR sensors for both AE and SE with a cumulative season reduction in DLI of 14% and a mean season reduction in DLI of 18% for AE and a cumulative season reduction in DLI of 21% and a mean season reduction in DLI of 21% for SE (**Figures 3A**, AE and **B**, SE). In AE, there was no significant PAR reduction for the first ~4 months of the season, while the remaining ~3 months of the season had significant reductions in DLI due to LBF. In SE, there was a significant reduction in PAR from transplanting for ~3.5 months, followed by ~2.5 months during winter with no significant reduction in DLI due to LBF, while the last ~1.5 months of the crop showed a significant LBF PAR reduction. Although AE and SE were roughly the same length, ~57% of the AE season’s growth was without light reduction due to LBF, whereas only ~33% of the SE season’s growth was without light reduction due to LBF.

### 3.2 PAR and net radiation are different at solar transition periods throughout AE and SE

Given that LBF reduces canopy PAR differentially throughout the year, it is important to understand how it impacts other light regions and the daily sums of PAR during solar transition periods. Solar azimuth, which is the location of sunrise and sunset on the horizon, and solar altitude, which is the height of the sun from the horizon, transition during the year with impacts on the length of the photoperiod (Bowen, 1979). These solar transition periods around the equinoxes and solstices may affect the light differentials produced by LBF and further influence the physiological responses of the crop.

The incoming SW radiation was significantly reduced (53%–58%) by LBF across solar transition periods and seasons (AE and SE). The largest reductions in incoming SW radiation by LBF for AE and SE were 60% and 58%, respectively, which occurred during the winter solstice in both seasons. The smallest reductions in incoming SW radiation by LBF for AE and SE were 54% and 52%, respectively, which occurred at the beginning of each experiment in both seasons. Although these reductions vary, the reduction in incoming SW radiation is relatively consistent across solar transition periods and seasons. However, the reduction due to LBF on outgoing SW radiation varied more than the incoming SW radiation, ranging from 31% to 70% across both seasons and solar transition periods. The largest reductions in outgoing SW radiation were 69% for AE at the summer solstice and 70% for SE at the Spring equinox, both of which occurred at the end of each crop cycle (**Table 2**).

**Table 2:** Incoming and outgoing SW and LW radiation for the start of the Autumn Experiment (AE) and the Summer Experiment (SE) and following solar transitions through which each crop was grown. Not significant p-values are indicated as "NS".

			Incoming SW					Outgoing SW				
Time of Year	Date	DAT	Control (kWh m^−2^ day^−1^)	LBF (kWh m^−2^ day^−1^)	Δ (kWh m^−2^ day^−1^)	Change (%)	*p*-Value	Control (kWh m^−2^ day^-1^)	LBF (kWh m^−2^ day^−1^)	Δ (kWh m^−2^ day^−1^)	Change (%)	*p*-Value
2019 Start AE	17-04-19	12	3.81 ± 0.07	1.73 ± 0.03	−2.08	−55	***	0.33 ± 0.01	0.23 ± 0.01	−0.10	−31	***
2019 Winter Solstice	19-06-19	75	2.33 ± 0.01	0.98 ± 0.01	−1.35	−58	***	0.16 ± 0.01	0.11 ± 0.01	−0.05	−31	***
2019 Spring Equinox	20-09-19	168	5.19 ± 0.14	2.30 ± 0.05	−2.89	−56	***	0.47 ± 0.02	0.22 ± 0.02	−0.26	−54	***
2019 Summer Solstice	29-11-19	238	6.15 ± 0.11	2.70 ± 0.06	−3.46	−56	***	0.76 ± 0.02	0.23 ± 0.01	−0.53	−69	***
2020 Start SE	01-03-20	44	5.40 ± 0.04	2.56 ± 0.03	−2.84	−53	***	0.48 ± 0.01	0.21 ± 0.01	−0.27	−56	***
2020 Autumn Equinox	19-03-20	62	4.95 ± 0.11	2.30 ± 0.05	−2.65	−54	***	0.43 ± 0.01	0.17 ± 0.02	−0.26	−60	***
2020 Winter Solstice	19-06-20	154	2.25 ± 0.01	0.96 ± 0.01	−1.29	−57	***	0.18 ± 0.01	0.09 ± 0.01	−0.09	−52	***
2020 Spring Equinox	15-09-20	242	4.67 ± 0.05	2.10 ± 0.02	−2.57	−55	***	0.56 ± 0.01	0.17 ± 0.01	−0.39	−70	***
			Incoming LW					Outgoing LW				
Time of Year	Date	DAT	Control (kWh m^−2^ day^−1^)	LBF (kWh m^−2^ day^−1^)	Δ (kWh m^−2^ day^−1^)	Change (%)	*p*-Value	Control (kWh m^−2^ day^−1^)	LBF (kWh m^−2^ day^−1^)	Δ (kWh m^−2^ day^−1^)	Change (%)	*p*-Value
2019 Start AE	17-04-19	12	10.20 ± 0.03	10.67 ± 0.04	0.47	+ 5	***	10.60 ± 0.02	10.55 ± 0.02	−0.05	−1	.
2019 Winter Solstice	19-06-19	75	9.59 ± 0.06	9.83 ± 0.03	0.24	+ 3	**	10.24 ± 0.02	10.18 ± 0.02	−0.06	−1	NS
2019 Spring Equinox	20-09-19	168	9.93 ± 0.04	10.63 ± 0.06	0.69	+ 7	***	10.42 ± 0.01	10.41 ± 0.02	−0.01	0	NS
2019 Summer Solstice	29-11-19	238	10.33 ± 0.04	11.19 ± 0.08	0.86	+ 8	***	10.58 ± 0.02	10.53 ± 0.02	−0.05	0	NS
2020 Start SE	01-03-20	44	10.57 ± 0.14	11.33 ± 0.12	0.76	+ 7	**	10.84 ± 0.10	10.69 ± 0.07	−0.16	−1	NS
2020 Autumn Equinox	19-03-20	62	10.39 ± 0.09	11.15 ± 0.09	0.76	+ 7	***	10.62 ± 0.04	10.52 ± 0.04	-0.10	-1	NS
2020 Winter Solstice	19-06-20	154	9.71 ± 0.06	10.09 ± 0.02	0.39	+ 4	***	10.11 ± 0.02	10.10 ± 0.01	-0.01	0	NS
2020 Spring Equinox	15-09-20	242	10.16 ± 0.10	10.85 ± 0.07	0.69	+ 7	***	10.31 ± 0.03	10.30 ± 0.02	-0.01	0	NS

Data are average daily sums across three full sun days around each respective Date ± standard error of the mean (n = 6) and statistical analysis was performed using parametric or non-parametric analyses (one-way analysis of variance (OA), Kruskal–Wallis (KW), or Welch**’**s ANOVA (WA). The p-values significance levels indicated as “**” (p-value <0.01), and “***” (p-value <0.001).

Interestingly, LBF increased incoming LW radiation by 3%–8% across both seasons. The smallest increases in incoming LW radiation were 3% for AE and 4% for SE, which occurred during the winter solstice in both seasons. Larger increases in incoming LW radiation occurred at the spring equinox for AE with an 8% increase, while the start of the season, the autumn equinox, and spring equinox all showed a 7% increase by LBF for SE. Outgoing LW radiation was not impacted by LBF (**Table 2**).

The LBF affected incoming and outgoing SW and LW radiation, as well as net SW (incoming SW − outgoing SW), net LW (incoming LW − outgoing LW), and net radiation (net SW + net LW). Net SW radiation was reduced by LBF across AE and SE and solar transition periods by 52%–60%, with the largest reductions occurring at the winter solstices for both AE and SE, with observed reductions of 60% and 58%, respectively (**Table 3**). LBF increased the net LW radiation by 47%–480% across both the AE and SE and solar transition periods. The smallest increases in net LW occurred during the winter solstice for both AE and SE; for these periods, net LW was negative. However, for all other solar transition periods for AE and SE, LBF caused net radiation to be positive. The largest increase in net LW was 371% during the summer solstice for AE and 480% during the spring equinox for SE; both periods were at the end of the crop season. Overall, LBF reduced net radiation across AE and SE and solar transition periods by 36%–66%, with the highest reductions in net radiation occurring on the winter solstices for both AE and SE, with reductions of 66% and 47%, respectively.

**Table 3:** Net SW, net LW and net radiation for the start of the Autumn Experiment (AE) and the Summer Experiment (SE) and following solar transitions through which each crop was grown.

Net SW							
Time of Year	Date	DAT	Control (kWh m^−2^ day^−1^)	LBF (kWh m^−2^ day^−1^)	Δ (kWh m^−2^ day^−1^)	Change (%)	*p*- Value
2019 Start AE	17-04-19	12	3.49 ± 0.06	1.51 ± 0.03	−1.98	−57	***
2019 Winter Solstice	19-06-19	75	2.17 ± 0.02	0.87 ± 0.01	−1.30	−60	***
2019 Spring Equinox	20-09-19	168	4.72 ± 0.13	2.08 ± 0.05	−2.63	−56	***
2019 Summer Solstice	29-11-19	238	5.39 ± 0.10	2.46 ± 0.06	−2.93	−54	***
2020 Start SE	01-03-20	44	4.92 ± 0.05	2.35 ± 0.02	−2.57	−52	***
2020 Autumn Equinox	19-03-20	62	4.52 ± 0.10	2.12 ± 0.04	−2.39	−53	***
2020 Winter Solstice	19-06-20	154	2.06 ± 0.02	0.87 ± 0.01	−1.19	−58	***
2020 Spring Equinox	15-09-20	242	4.11 ± 0.05	1.93 ± 0.01	−2.18	−53	***
Net LW							
Time of Year	Date	DAT	Control (kWh m^−2^ day^−1^)	LBF (kWh m^−2^ day^−1^)	Δ (kWh m^−2^ day^−1^)	Change (%)	*p*-Value
2019 Start AE	17-04-19	12	−0.40 ± 0.02	0.12 ± 0.03	0.52	+ 131	***
2019 Winter Solstice	19-06-19	75	−0.65 ± 0.03	−0.35 ± 0.02	0.31	+ 47	***
2019 Spring Equinox	20-09-19	168	−0.49 ± 0.03	0.22 ± 0.04	0.71	+ 145	***
2019 Summer Solstice	29-11-19	238	−0.24 ± 0.03	0.66 ± 0.07	0.91	+ 371	***
2020 Start SE	01-03-20	44	−0.27 ± 0.04	0.64 ± 0.05	0.92	+ 334	***
2020 Autumn Equinox	19-03-20	62	−0.23 ± 0.05	0.62 ± 0.05	0.86	+ 368	***
2020 Winter Solstice	19-06-20	154	−0.40 ± 0.05	−0.01 ± 0.01	0.40	+ 99	***
2020 Spring Equinox	15-09-20	242	−0.14 ± 0.07	0.55 ± 0.06	0.69	+ 480	***
Net Radiation							
Time of Year	Date	DAT	Control (kWh m^−2^ day^−1^)	LBF (kWh m^−2^ day^−1^)	Δ (kWh m^−2^ day^−1^)	Change (%)	*p*- Value
2019 Start AE	17-04-19	12	3.09 ± 0.08	1.63 ± 0.05	−1.45	−47	***
2019 Winter Solstice	19-06-19	75	1.52 ± 0.04	0.52 ± 0.03	−0.99	−66	***
2019 Spring Equinox	20-09-19	168	4.23 ± 0.11	2.30 ± 0.06	−1.93	−46	***
2019 Summer Solstice	29-11-19	238	5.15 ± 0.10	3.13 ± 0.10	−2.02	−39	***
2020 Start SE	01-03-20	44	4.64 ± 0.07	2.99 ± 0.07	−1.65	−36	***
2020 Autumn Equinox	19-03-20	62	4.28 ± 0.06	2.75 ± 0.05	−1.54	-36	***
2020 Winter Solstice	19-06-20	154	1.61 ± 0.05	0.86 ± 0.01	−0.75	−47	***
2020 Spring Equinox	15-09-20	242	3.97 ± 0.09	2.48 ± 0.07	−1.49	−37	***

Data are average daily sums across three full sun days around each respective Date ± standard error of the mean (n = 6) and statistical analysis was performed using parametric or non-parametric analyses (one-way analysis of variance (OA), Kruskal–Wallis (KW), or Welch’s ANOVA (WA). Significance indications are stated in the methodology section.

While roof-level PAR showed a consistent reduction in DLI across both AE and SE, canopy-level PAR sensors only showed a significant reduction in DLI during the high light period when comparing data using a smoothed plot representation. However, when comparing full sun daily sums during solar transition periods, LBF reduced DLI significantly, and this reduction seems to be influenced by the height of the canopy PAR sensors (**Table 4**). LBF reduced the canopy level DLI by 8%–25.3%, with a span of 17.3%, across solar transition periods and seasons. The largest reduction in AE was 23.3% in the summer solstice and 25.3% in the SE at the autumn equinox. Roof-level PAR sensors showed a more consistent reduction in DLI across solar transition periods, for both AE and SE, with a span of 4.2%, ranging from 26.0% to 30.2%.

**Table 4:** DLI (mol m−2 day−1) of photosynthetically active radiation average across canopy and roof level PAR sensors for the beginning of each experiment and solar transition periods for the Autumn Experiment (AE) and the Summer Experiment (SE).

PAR at Canopy							
Time of Year	Date	DAT	Control (mol m^−2^ day^−1^)	LBF (mol m^−2^ day^−1^)	Δ (mol m^−2^ day^−1^)	Change (%)	*p*-Value
2019 Beginning of AE	17-04-19	12	15.4 ± 0.4	13.6 ± 0.2	−1.7	−11.2	**
2019 Winter Solstice	19-06-19	75	8.8 ± 0.2	8.1 ± 0	−0.7	−8.0	**
2019 Spring Equinox	20-09-19	168	22.2 ± 0.8	18.6 ± 0.5	−3.5	−15.8	**
2019 Summer Solstice	29-11-19	238	26.7 ± 0.8	20.5 ± 0.7	−6.2	−23.3	***
2020 Beginning of SE	01-03-20	44	25.2 ± 0.2	19.3 ± 0.2	−5.9	−23.4	***
2020 Autumn Equinox	19-03-20	62	22.4 ± 0.4	16.7 ± 0.2	−5.7	−25.3	***
2020 Winter Solstice	28-06-20	154	9.9 ± 0.2	8.2 ± 0.2	−1.7	−16.7	***
2020 Spring Equinox	15-09-20	242	0.2 ± 0	0.3 ± 0	0.1	53.7	***
PAR at Roof							
Time of Year	Date	DAT	Control (mol m^−2^ day^−1^)	LBF (mol m^−2^ day^−1^)	Δ (mol m^−2^ day^−1^)	Change (%)	*p*-Value
2019 Beginning of AE	17-04-19	12	26.8 ± 0.3	19.5 ± 0.3	−7.3	−27.2	***
2019 Winter Solstice	19-06-19	75	15.2 ± 0.1	10.8 ± 0.1	−4.4	−29.2	***
2019 Spring Equinox	20-09-19	168	34.6 ± 0.7	25.3 ± 0.6	−9.3	−26.9	***
2019 Summer Solstice	29-11-19	238	39.5 ± 0.9	29.1 ± 0.7	−10.4	−26.3	***
2020 Beginning of SE	01-03-20	44	38 ± 0.4	28.1 ± 0.3	−9.9	−26.0	***
2020 Autumn Equinox	19-03-20	62	33.8 ± 0.6	24.7 ± 0.5	−9.2	−27.1	***
2020 Winter Solstice	28-06-20	154	14.9 ± 0.1	10.6 ± 0.3	−4.3	−28.7	***
2020 Spring Equinox	15-09-20	242	31.1 ± 0.2	21.7 ± 0.2	−9.4	−30.2	***

Data are average daily sums across three full sun days around each respective Date ± standard error of the mean (n = 6) and statistical analysis was performed using parametric or non-parametric analyses (one-way analysis of variance (OA), Kruskal–Wallis (KW), or Welch’s ANOVA (WA). Significance indications are stated in the methodology section.

### 3.3 LBF film increases diffuse light conditions through AE and SE

**Figure 4:**
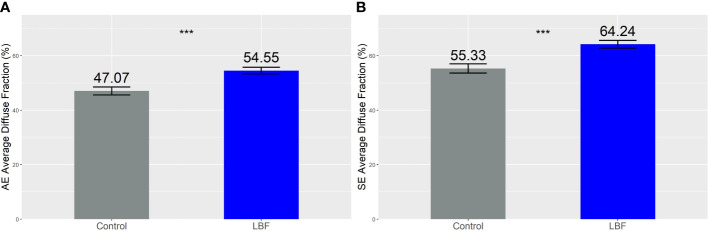
Average diffuse fraction (%) across the whole season for the **(A)** Autumn Experiment (AE) and **(B)** Summer Experiment (SE). The numbers above the bars are the diffuse light fraction (%), and the error bars are the standard error of the mean. Both seasons were significantly different across the control and LBF groups, *** indicates P <0.001 from a one-way analysis of variance.

**Figure 5:**
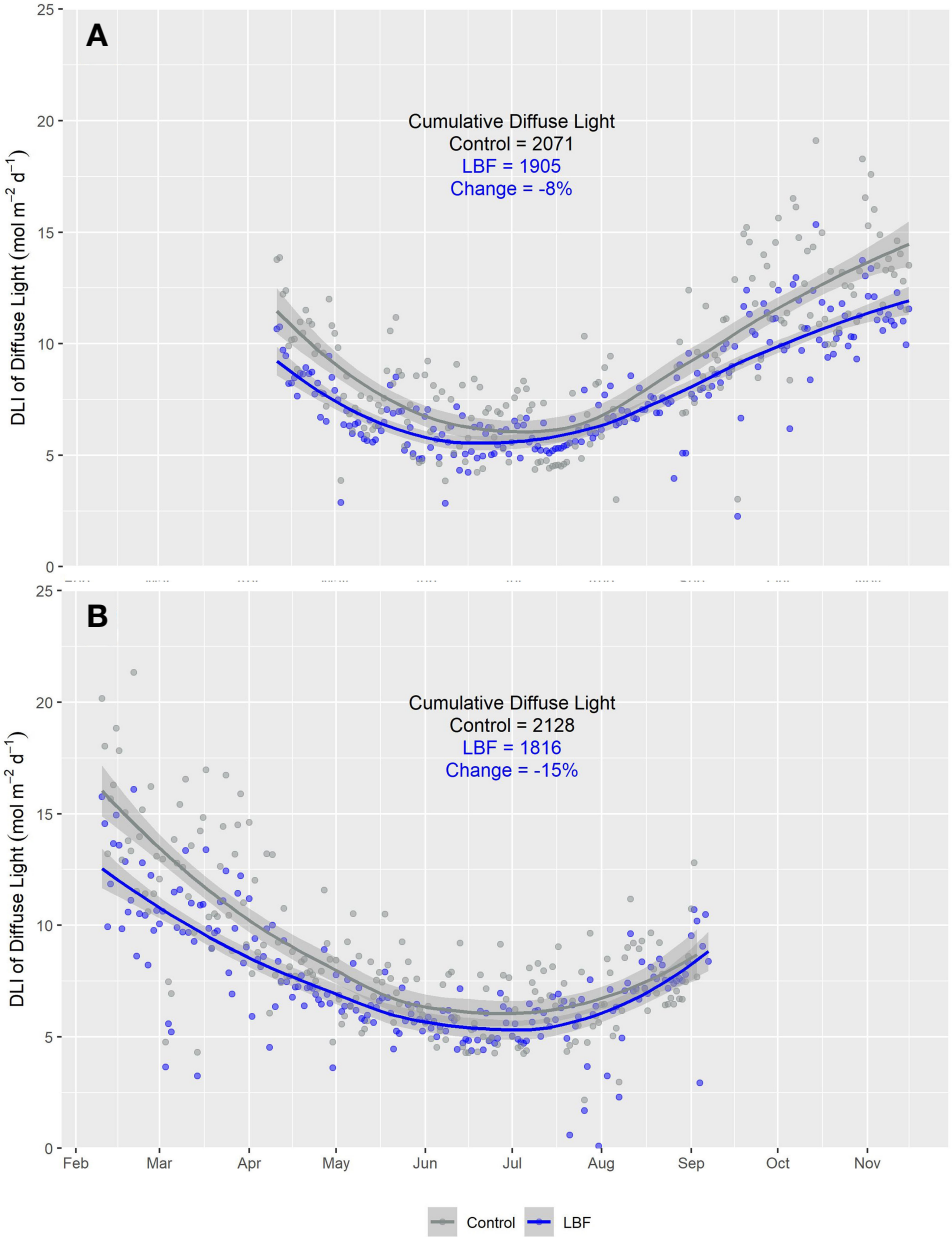
Smooth plot of the cumulative daily light integral (DLI; mol m^−2^ d^−1^) of diffuse light over time for both LBF and control for **(A)** AE and **(B)** SE. Each data point represents the average DLI for a single day. Each data point represents the average DLI from five sensors for a single day. The blue (LBF) and gray (control) lines are fitted loess curves with formula y ~ x and shaded regions represent the 95% confidence interval.

The daily average proportion of diffuse light was increased by LBF in AE (**Figure 4A**) and SE (**Figure 4B**). In AE, the average daily diffuse light fractions in the control and LBF groups were 47.0% and 54.5%, respectively. In SE, the average daily diffuse light fractions were 55.3% and 64.2% in the control and LBF groups, respectively. Although LBF generated a higher diffuse fraction of light, the total diffuse light received was significantly less than that of the control, except from June to mid-August for AE (**Figure 5A**) and from May to September for SE when there was no impact (**Figure 5B**).

### 3.4 LBF significantly reduces far-red light throughout canopy profile

**Figure 6:**
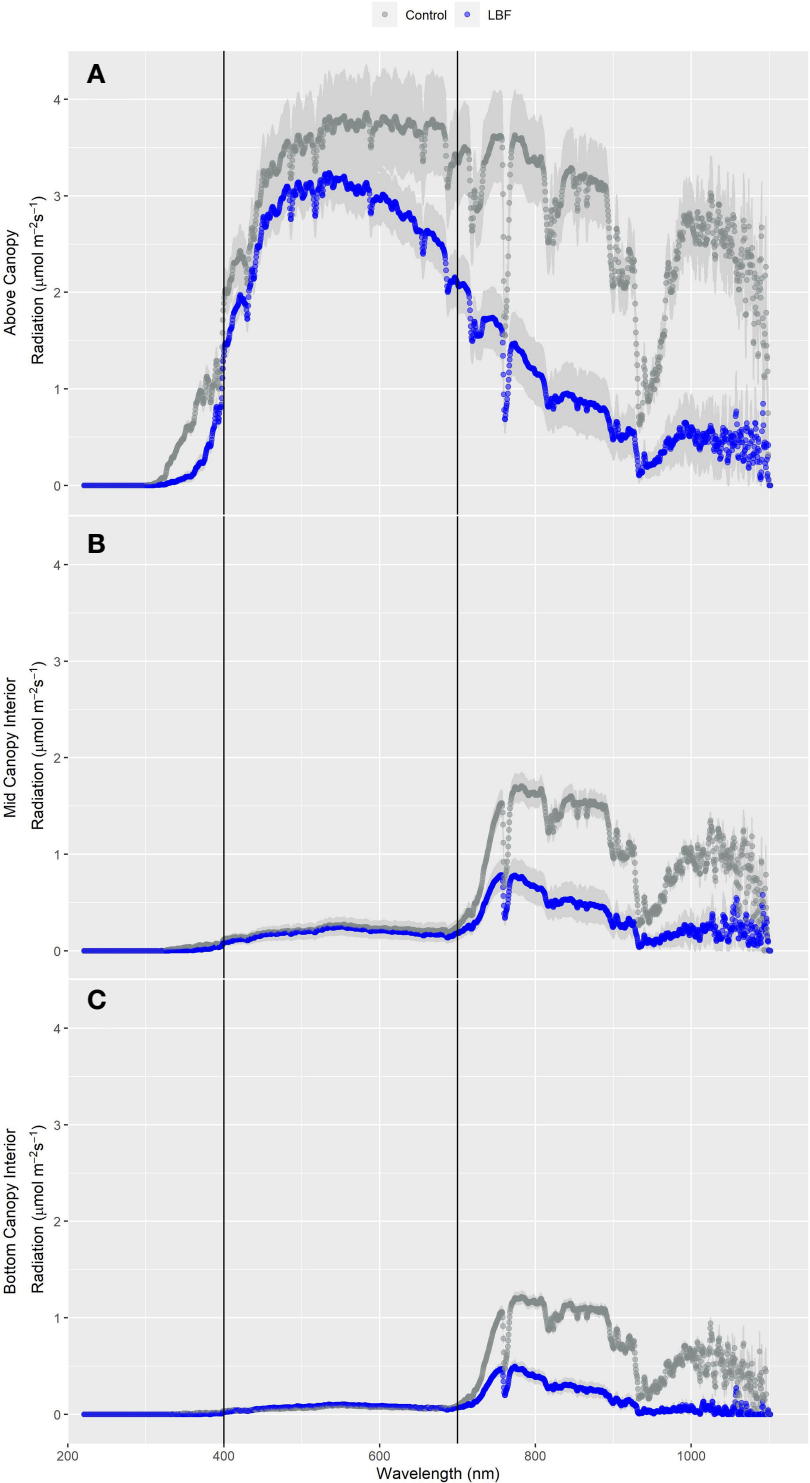
Light spectrum measured just above the canopy **(A)**, mid-height within the canopy **(B)**, and below the canopy **(C)** on 17-09-2020 for the SE crop during full sun conditions. For each spectrum, each point is the measured µmol/m^2^/s every 0.5 nm across the 300 nm–1,100 nm sensor range. The shaded regions represent the standard error of the mean.

Diffuse light penetrates deeper into the canopy than direct light, potentially improving photosynthesis in mid- and bottom-canopy leaves (Babla et al., 2020). Above-canopy light was higher overall for the control than for LBF (**Figure 6A**), with a significant reduction of 60% in far-red light (P = 0.004, **Table 5**). Minimal light was transmitted to mid-canopy (**Figure 6B**) and low-canopy (**Figure 6C**) heights in the blue, green, red, and PAR spectral regions with no significant difference between LBF and control; however, LBF significantly reduced far-red light for mid-canopy and low-canopy positions by 57% (P = 0.002) and 64% (P = 1.01 × 10^−5^), respectively (**Table 5**).

**Table 5:** Light region average sums for each position throughout the canopy.

Above Canopy					
Wavelength (nm)	Control (µmol m^−2^ s^−1^)	LBF (µmol m^−2^ s^−1^)	Δ (µmol m^−2^ s^−1^)	Change (%)	*p*-value
Blue (340 nm–499 nm)	676.2 ± 76.9	523.0 ± 44.7	−153	−22.7	0.078
Green (500 nm–599 nm)	732.2 ± 92.5	612.1 ± 49.5	−120.00	−16.4	0.109
Red (600 nm–699 nm)	726.7 ± 97.0	524.1 ± 55.3	−203	−27.9	0.078
Far-red (719 nm–850 nm)	834.5 ± 107.5	331.8 ± 77.5	−503	−60.2	0.007
PAR (380 nm–699 nm)	2085.6 ± 262.0	1652.8 ± 145.2	−433	−20.8	0.078
Mid Canopy					
Wavelength (nm)	Control(µmol m^−2^ s^−1^)	LBF(µmol m^−2^ s^−1^)	Δ (µmol m^−2^ s^−1^)	Change (%)	*p*-value
Blue (340 nm–499 nm)	40.3 ± 11.8	32.9 ± 12.3	−7	−18.3	0.262
Green (500 nm–599 nm)	48.3 ± 14.7	44.7 ± 15.7	−4	−7.5	0.337
Red (600 nm–699 nm)	43.2 ± 15.4	36.3 ± 14.0	−7	−16.1	0.200
Far-red (719 nm–850 nm)	353.7 ± 30.6	153.7 ± 38.2	−200	−56.5	0.016
PAR (380 nm–699 nm)	128.8 ± 41.2	113.5 ± 41.8	−15	−11.9	0.262
Bottom Canopy					
Wavelength (nm)	Control(µmol m^−2^ s^−1^)	LBF(µmol m^−2^ s^−1^)	Δ (µmol m^−2^ s^−1^)	Change (%)	*p*-value
Blue (340 nm–499 nm)	12.0 ± 2.7	11.7 ± 2.2	−0.3	−2.1	0.943
Green (500 nm–599 nm)	16.6 ± 3.1	18.4 ± 2.9	1.8	11.1	0.749
Red (600 nm–699 nm)	13.9 ± 3.2	14.4 ± 2.9	0.6	4.1	0.631
Far-red (719 nm–850 nm)	246.4 ± 12.7	89.6 ± 14.5	−157.00	−63.7	0.004
PAR (380 nm–699 nm)	41.8 ± 8.7	44.6 ± 8.0	3	6.7	0.873

Data are mean ± standard error of the mean (n = 6) and statistical analysis was performed using a Kruskal–Wallis Test. Far-red radiation is significantly reduced by LBF throughout the canopy.

### 3.5 Plant morphological responses to LBF and seasons

Plant growth rate, number of buds, flowers, developing fruit, yield (fruit mass per plant and fruit number per plant), and pruned biomass were measured in both experiments (**Table 6**). There was no difference in growth rate across LBF and the control for the red variety in AE and both varieties in SE; however, the growth rate was significantly higher in SE than in AE (red variety, P = 4.7 × 10^−14^ and orange variety, P = 2 × 10^−16^). The growth rate was slightly increased by LBF in the orange variety of AE. During AE, LBF the increased numbers of buds for both red and orange capsicum by 11.2% (P = 0.006) and 16.4% (P = 1.9 × 10^−4^), flowers for both red and orange varieties by 16.1% (P = 4.9 × 10^−4^) and 13.8% (P = 4.0 × 10^−4^), respectively, and developing fruit for the orange variety only by 8% (P = 0.015). There was no treatment effect for buds, flowers, or developing fruit in SE; however, SE plants produced more buds, flowers, and developing fruit than in AE. There was significantly more pruned biomass in the AE season than in the SE for both varieties (red: P = 7.4 × 10^−9^ and orange: P = 3.5 × 10^−6^); however, there was no difference in pruned biomass across the control and LBF for either AE or SE.

**Table 6:** Summary of statistical analyses using one-way and two-way analysis of variance (ANOVA) for the LBF and experiment effect on growth and yield parameters.

Parameter	Exp	Red							Orange						
		Control	LBF	Change(%)	LBF *p*-value	LBF	Exp *p*-value	LBF*Exp	Control	LBF	Change(%)	LBF *p*-value	LBF	Exp *p*-value	LBF*Exp
Growth parameters															
Growth rate(cm day^−1^)	AE	0.83 ± 0.02	0.85 ± 0.02	2.4	0.605	0.515	**<2 × 10** ^−^ **^16^**	0.201	0.77 ± 0.02	0.83 ± 0.02	7.8	**0.015**	0.552	**<2 × 10** ^−^ **^16^**	**0.004**
	SE	1.53 ± 0.04	1.47 ± 0.03	−3.9	0.262				1.49 ± 0.04	1.39 ± 0.04	−0.7	0.06			
Buds(n/plant/week)	AE	10.90 ± 0.36	12.12 ± 0.25	11.2	**0.006**	**0.03**	**<2 × 10** ^−^ **^16^**	0.614	10.84 ± 0.26	12.62 ± 0.37	16.4	**1.9 × 10** ^−^ **^4^**	**8.7 × 10^-4^**	**<2 × 10** ^−^ **^16^**	0.207
	SE	17.42 ± 0.59	18.18 ± 0.53	4.4	0.342				17.47 ± 0.44	18.28 ± 0.43	4.6	0.189			
Flower(n/plant/week)	AE	1.37 ± 0.04	1.59 ± 0.04	16.1	**4.9 × 10** ^−^ **^4^**	0.104	**<2 × 10** ^−^ **^16^**	**0.015**	1.67 ± 0.04	1.90 ± 0.05	13.8	**4.0 × 10** ^−^ **^4^**	**0.009**	0.258	**0.022**
	SE	2.02 ± 0.06	1.97 ± 0.06	−2.5	0.623				1.83 ± 0.05	1.84 ± 0.05	0.5	0.821			
Developing Fruit (n/plant/week)	AE	6.40 ± 0.17	6.53 ± 0.13	2.0	0.537	0.71	**4.4 × 10** ^−^ **^4^**	0.341	6.13 ± 0.14	6.62 ± 0.14	8.0	**0.015**	0.65	**2.2 × 10** ^−^ **^7^**	**0.009**
	SE	7.42 ± 0.30	7.12 ± 0.26	−4.0	0.454				5.70 ± 0.19	5.35 ± 0.15	−6.1	0.162			
Pruned biomass*(g/plant/season)	AE	50.89 ± 1.45	48.82 ± 0.91	−4.1	0.271	0.673	**7.4 × 10** ^−^ **^9^**	0.346	48.68 ± 2.34	48.78 ± 0.97	0.2	0.970	0.4	**3.5 × 10** ^−^ **^6^**	0.378
	SE	28.69 ± 1.13	29.49 ± 2.09	2.8	0.747				32.32 ± 2.69	28.22 ± 2.71	−12.7	0.324			
Harvested fruit yield and marketability															
Plant Yield (kg/plant/season)	AE	2.48 ± 0.06	2.36 ± 0.07	−4.8	0.197	**5.62 × 10^-8^**	**1.17 × 10** ^−^ **^4^**	**6.08 × 10** ^−^ **^5^**	2.17 ± 0.05	2.21 ± 0.04	2.1	0.51	**0.005**	**1.02 × 10** ^−^ **^9^**	**2.43 × 10** ^−^ **^4^**
	SE	2.37 ± 0.09	1.67 ± 0.08	−29.4	**8.58 × 10** ^−^ **^9^**				2.03 ± 0.05	1.70 ± 0.06	−16.7	**2.64 × 10** ^−^ **^5^**			
Plant Fruit Number (n/plant/season)	AE	13.26 ± 0.32	11.84 ± 0.29	−10.7	**0.001**	**1.85 × 10^-11^**	**3.99 × 10** ^−^ **^14^**	**0.005**	13.54 ± 0.35	13.05 ± 0.26	−3.7	0.249	**8.62 × 10^-5^**	**<2 × 10** ^−^ **^16^**	**0.022**
	SE	11.51 ± 0.40	8.19 ± 0.34	−28.9	**2.25 × 10** ^−^ **^9^**				10.65 ± 0.30	8.77 ± 0.28	−17.6	**1.07 × 10** ^−^ **^5^**			
Marketable Fruit Yield (kg/plant/season)	AE	2.17 ± 0.06	2.12 ± 0.06	−2.2	0.552	**4.15 × 10^-7^**	**0.004**	**1.08 × 10** ^−^ **^5^**	1.88 ± 0.05	2.03 ± 0.04	8.4	**0.016**	0.082	**6.01 × 10^-4^**	**3.14 × 10** ^−^ **^4^**
	SE	2.28 ± 0.08	1.61 ± 0.07	−29.3	**1.68 × 10** ^−^ **^8^**				1.94 ± 0.06	1.60 ± 0.06	−17.5	**5.75 × 10** ^−^ **^5^**			
Marketable Fruit Number (n/plant/season)	AE	10.60 ± 0.28	9.98 ± 0.25	−5.8	0.104	**4.71 × 10^-6^**	**1.53 × 10** ^−^ **^14^**	**0.004**	10.52 ± 0.28	10.96 ± 0.22	4.1	0.223	0.102	**<2 × 10** ^−^ **^16^**	**0.002**
	SE	8.81 ± 0.46	6.23 ± 0.34	−29.3	**1.33 × 10** ^−^ **^5^**				7.81 ± 0.32	6.45 ± 0.31	−17.4	**0.002**			

The values are mean ± standard error of mean (n = 4–40). Change represents (LBF − Control/Control) × 100. For yield and fruit measurements, a season is equal to 248 days which is the length of time between first harvest and last harvest for SE. AE was multiplied by 1.05 to be equivalent to the season length of SE. * is dry mass of pruned biomass. Values in bold text highlight p-values < 0.05.

### 3.6 LBF mainly reduces fruit yield in SE and not in AE

The total fruit yield per m^2^ per year was higher for AE than for SE for both varieties (**Table 6**). During AE, the red variety produced 12.55 kg m^−2^ in control and 11.95 kg m^−2^ in LBF, and the orange variety produced 10.98 kg m^−2^ in control and 11.21 kg m^−2^ in LBF. During SE, the red variety produced 12.00 kg m^−2^ in control and 8.47 kg m^−2^ in LBF, and the orange variety produced 10.31 kg m^−2^ in control and 8.59 kg m^−2^ in LBF. LBF caused a slight increase in yield for the orange variety with an AE of 2.1%; however, in SE, LBF produced a 16.7% reduction in the orange variety. LBF significantly reduced plant fruit numbers for the red variety for both AE and SE by 15% (P = 0.003) and 31.5% (P = 1.1 × 10^−9^), respectively. The fruit number per plant for the orange variety showed no difference across LBF and control for AE; however, there was an 18.1% reduction in fruit number in LBF in comparison to the control in SE (P = 6.4 × 10^−15^). When comparing marketable yield mass, LBF increased the orange variety by only 8.4% (P = 0.016), while the read variety was unaffected. However, marketable fruit numbers were not affected by LBF in AE. In SE, the yield mass was decreased by LBF for red and orange by 29.3% (P = 1.68 × 10^−8^) and 17.5% (P = 5.75 × 10^−8^), respectively. Marketable fruit number was also reduced by LBF by similar proportions as mass, with a reduction in the red variety of 29.3% (P = 1.33 × 10^−5^) and a reduction in the orange variety of 17.4% (P = 0.002).

## 4 Discussion

This experimental section provided an analysis of the impact of LBF on light quantity and quality throughout two crop cycles of *C. annuum* planted in the autumn of 2019 (AE) and in the summer of 2020 (SE) and the subsequent biological responses of growth, biomass, and fruit yield. Overall, the impact of LBF and planting time differentially affected crop growth and productivity.

### 4.1 Impacts of LBF on light environment across all installed light sensors

While the smoothed plot for canopy-level PAR sensors did not show an impact from LBF during winter months and during lower sensor height positions for AE and SE, the sums of full sun days around the winter solstice showed a significant reduction in LBF compared to the control. When full sun days were compared, canopy level PAR was reduced differentially depending on the time of year for both AE and SE, ranging from 8% to 25.3%, whereas roof level reduction remained constant (26.0%–30.2%) regardless of the time of year. This suggests that the canopy light environment is dependent on crop height. Structural shading impacts light measurements such that higher canopy positions have less shading overall than lower positions, particularly during winter months when shadows are longer. Inside a glasshouse, structural shading can reduce light by up to 30% (Gruda, 2005). Differences in the structural components and the directional position of the glasshouse may cause specific light sensors to be more shaded than others, even though they are in the same position in each room. PAR reduction differences may also occur over the year because some of the glass panels on the wall over the door and on the roof close to the eaves could not be covered with LBF owing to infrastructure. As the sun angle decreases during the winter months, light begins to enter the room from positions that do not have LBF applied, whereas with high sun angles in summer, light primarily enters from the roof, which is fully covered by LBF. Further investigation of canopy-level light differences across room areas with height would be useful.

Net radiation measurements are integral to understanding the energy balance of a system, and net radiometers are used in natural ecosystems, broad acre cropping, and PC agriculture (Koksal et al., 2018; Rebmann et al., 2018; Saadon et al., 2021). Introducing film technology to a glasshouse that reflects and absorbs SW light will impact the energy balance of the crop and the requirements to maintain optimal crop temperatures. In this study, it was found that while incoming SW radiation was reduced relatively consistently throughout the year (53%–58%), the outgoing SW and incoming LW varied with solar transition periods, and outgoing LW was unimpacted by LBF. The largest reduction in outgoing SW radiation was at the end of the summer solstice for AE and the spring equinox for SE. During this period, the top canopy of the crop absorbs larger amounts of SW radiation, including PAR and some LW radiation, because the overall leaf area is highest at the end of the crop cycle (Rosati et al., 2001). The incoming LW radiation increased overall for LBF rooms, with the largest increase in the summer (7%) and the lowest increase in the winter (3%). The larger increase in incoming LW radiation during the summer was due to the LBF absorbing the blocked SW radiation as heat and then emitting it into the room. However, SW radiation accounts for the majority of energy incident to the glasshouse, and the overall net radiation across AE, SE, and solar transition periods was reduced by 36%–66% in both seasons. The LBF was designed for residential buildings with a vertical orientation to the sun and was not designed for rooftops. While a primarily horizontal orientation to the sun may facilitate heat absorption and negatively affect the cooling capacity of the rooms (Chaiyapinunt et al., 2005), the total amount of net radiation reduction by LBF should result in a reduced need for cooling to maintain optimal temperatures throughout high radiation months. To understand the energy balance of LBF and control better, temperature measurements of the roof, walls, and canopy at different heights are required.

Light penetration throughout the canopy is an important factor for growers because overall photosynthesis increases when more of the canopy profile is illuminated. Growers base planting density on light levels throughout the year, and planting density and diffuse light levels affect light transmission throughout the crop canopy (Jovicich et al., 2004; Marcelis et al., 2006). LBF increased the diffuse fraction of total PAR; however, total diffuse light was either not affected or slightly reduced by LBF. The impact of light transmission throughout the crop canopy profile was investigated, as the diffuse fraction of light was different across the LBF and control. There was no difference in the amount of blue, green, or red light (PAR) transmitted through the canopy profile between LBF and control, but far-red and NIR light were greatly reduced. Far-red light from 700 nm to 740 nm has been shown to increase photosynthesis (Zhen and Bugbee, 2020), which may lead to lower rates of photosynthesis in the lower canopy in LBF. However, non-photosynthetically active NIR light may result in a hotter canopy in the control than in the LBF, making optimal temperatures in the control during high radiation months harder to maintain.

### 4.2 Change in biological response under LBF and photoperiod across seasons

While capsicum is known to be a non-photoperiod-sensitive crop in regard to flowering, capsicums have high light requirements, requiring DLIs of 12 mol m^−2^ d^−1^ –30 mol m^−2^ d^−1^ for good productivity, and <12 mol m^−2^ d^−1^ is considered to be light-limited conditions (Cossu et al., 2020; Morgan, 2021). AE was transplanted in autumn under sufficient light conditions, but shortly after planting, the DLI dropped into light-limited conditions, which continued for the first ~4 months of the season with no reduction in DLI due to LBF. The last ~3 months of AE occurred under sufficient light conditions, with a significant reduction in DLI due to LBF. SE was transplanted and established under sufficient DLI, with reductions due to LBF for the first ~3.5 months of growth, then transitioned into light-limited conditions without a reduction in DLI due to LBF for a ~2.5-month period. The final ~1.5 months of the SE season occurred under sufficient light conditions, with a significant reduction due to LBF. Although both AE and SE had similar crop season lengths, ~57% of AE growth occurred under light-limited conditions with no reduction due to LBF, whereas only ~33% of SE growth occurred under light-limited conditions with no reduction due to LBF. LBF DLI levels during SE were also considered light-limited for almost the entire season.

Differences across seasons in light limitation, photoperiod, and reduction in DLI due to LBF at different crop developmental stages impacted crops in different ways. Photoperiod sensitivity is well documented in plant developmental progression, and photoperiod manipulation is used to increase yield by managing supplementary lighting and the timing of planting and harvesting for certain crops (Demers and Gosselin, 1999; Demers & Gosselin, 2002; Garcia and Lopez, 2020). For instance, assimilate partitioning between vegetative and reproductive structures is directly affected by the post-flowering photoperiod in soybean and further influences nodes per plant, which is directly related to seed pod production and thus greatly affects yield (Nico et al., 2019). Plant growth and fruit yield generally increase with photoperiod and DLI maxima, and longer photoperiods with the same DLIs have been shown to increase germination and improve flower growth (Elkins and Iersel, 2020). Extended photoperiods via supplemental lighting have been shown to significantly increase the fruit yield of *C. annuum* (Dorais et al., 1996). However, this increase in production depends on the photoperiod at specific times during the plant developmental cycle, and crops behave differently depending on whether the photoperiod is ascending or descending (Heuvelink, 1995; Dorais et al., 1996; Elkins and Iersel, 2020). In this study, differences in seasonal light across AE and SE generated differences in all biological parameters measured, including the number of buds, flowers developing fruit per plant, growth rate, pruned biomass, and yield.

Overall, the stem growth rate was affected by planting time, with SE exhibiting a faster growth rate than AE; however, LBF did not affect the growth rate for either AE or SE for the red variety or SE for the orange variety. However, LBF generated a marginal increase in the growth rate of the orange variety in the AE. The sufficient light environment during the transplant date in the summer for SE provided an abundant resource for stem elongation, as opposed to the light-limited planting date in the autumn for AE (Tang et al., 2019). A lower overall PAR in LBF could cause LBF to grow faster and taller. However, because LBF’s spectral qualities do not increase the R:FR ratio, the plants did not respond with a shade avoidance strategy (etiolation) (Hückstädt et al., 2013).

*C. annuum* varieties produce buds, flowers, and fruits in a cyclical pattern, and abortion of these organs is common, even when grown in a climate-controlled high-tech glasshouse (Wubs et al., 2009). Different factors influence the presence/abortion of these organs, with the main contributing factors being the light intensity, quality, and photoperiod. LBF increased the presence of buds and flowers in both varieties and developed fruit for the orange variety for AE only. LBF had no impact on the presence of buds, flowers, or fruit for SE, which may be due to the day neutrality of flower induction for this species under sufficient light conditions (Kristiansen and Andersen, 1993). The increase in these components during AE may be due to the slight decrease in the red to blue light ratio by LBF in the absence of canopy-level PAR reduction by LBF in a light-limited environment (Yang et al., 2022). While LBF only had an impact on AE for these traits, there was a significant increase in buds, flowers, and developing fruits for both varieties in SE compared with AE. Plants in the SE grew in higher light periods, with much of the primary production period occurring at a threshold or over sufficient DLI conditions for capsicum. Therefore, plants have adequate resources to produce more buds, flowers, and fruits (Tang et al., 2019; Morgan, 2021).

*C. annuum* is a non-photoperiod-sensitive species (Yang et al., 2017; Tang et al., 2019); however, there have been mixed findings on leaf area across varied photoperiods. Yamamoto et al. (2008) found no effect on leaf area in response to photoperiod; however, Elkins and Iersel (2020) showed that in response to lower PPFD, plants have been shown to increase leaf size. Although we did not measure leaf area directly, AE had more pruned biomass than SE, suggesting that vegetative growth and leaf area were higher for AE. Plants in AE started developing under light-limited conditions and may have increased leaf area to compensate for low light conditions and reduced photosynthesis per unit leaf area (Rylski and Spigelman, 1986; Rosati et al., 2001; Díaz-Pérez, 2013). Different pruning strategies are employed for different seasonal planting dates to maximize yield (Alsadon et al., 2013; Parniani et al., 2022). For our purposes, pruning regimens were the same across each season, which may have put one season at a yield advantage over the other.

The initial sufficient light environment for SE provided ample light for growth; however, seedling establishment for capsicum was more successful at lower DLI, thus providing a head start on fruit maturation for AE (Yang et al., 2017; Tang et al., 2019). The sufficient light environment for seedling establishment during SE may have unbalanced the plants for reproductive growth, thus producing a lower overall yield compared to AE. In the SE, LBF reduced the DLI season mean (21%) and yield (red: 29%; orange: 16%), which is supported by Marcelis et al. (2006), who found that every 1% reduction in DLI resulted in a ~1% reduction in yield. However, the yield was not affected by LBF in AE, even though the DLI season mean was reduced (18%). The yield was marginally higher for the orange variety in AE (2.1%); however, this marginal increase in yield can be considered no change. The yield reduction in SE seems to be related to light-limited thresholds for capsicum. For instance, during AE, DLI reduction by LBF started when both control and LBF were within sufficient light conditions, while during SE, in periods when LBF reduced DLI, LBF was either at threshold or in light-limited conditions, while the control was under sufficient light conditions. This differential impact of LBF across AE and SE is likely due to the combination of the (1) Initial light environmental conditions during seedling establishment, (2) amount of time each crop spent with significant light reduction due to LBF, and (3) the amount of time that the control was under sufficient light conditions, while the LBF was at or below the threshold.

## 5 Conclusion

Sensor technology is an important component of PC agriculture for improving crop growth and maximizing yield. The current study demonstrates the use of light sensors (e.g., net radiometer, diffuse light sensor, spectro-radiometer, and PAR sensors) to understand the energy balance, changes in light quantity, and light quality. We assessed the impact of a residential building film (LBF) on light quantity and quality, and subsequently on the growth and yield response of *C. annuum* L. LBF reduced light quantity and altered light quality. These changes in light environment had differential impacts on crop development and yield of *C. annuum* depending on the planting time, the amount of time the crop grew during light-limited versus sufficient light conditions, and whether LBF reduced DLI.

We conclude that (1) the sensors used in the current study were able to characterize the light quantity, light quality, and light energy balance that contribute to heat generation in glasshouses; (2) LBF reduces total net radiation but increases LW radiation which may contribute to heat load and negatively impact cooling capacity; (3) LBF was not appropriate for year-round capsicum production because it reduced yield in SE despite potential reductions in energy use; (4) LBF may be useful for producing crops during the high radiation months of the year; and (5) for future assessment of LBF and agricultural cover materials aimed at reducing energy usage, the additional measurement of both roof and crop canopy temperature should be used to further inform the energy balance within a PC growing environment.

## Data availability statement

The original contributions presented in the study are included in the article/supplementary files, further inquiries can be directed to the corresponding author.

## Author contributions

CM: Conceptualization, Data curation, Formal analysis, Investigation, Project administration, Visualization, Writing – original draft. SC: Conceptualization, Methodology, Supervision, Writing – review & editing. NK: Project administration, Writing – review & editing. WL: Project administration, Writing – review & editing. CC: Conceptualization, Funding acquisition, Writing – review & editing. OG: Funding acquisition, Writing – review & editing. Z-HC: Funding acquisition, Supervision, Writing – review & editing. DT: Funding acquisition, Supervision, Writing – review & editing.
